# Two valid and reliable tests for monitoring age-related memory performance and neophobia differences in dogs

**DOI:** 10.1038/s41598-022-19918-7

**Published:** 2022-09-28

**Authors:** Patrizia Piotti, Andrea Piseddu, Enrica Aguzzoli, Andrea Sommese, Eniko Kubinyi

**Affiliations:** 1 Department of Ethology, ELTE Eötvös Loránd University, Pázmány Péter sétány 1/c, 1117 Budapest, Hungary; 2 Department of Veterinary Medicine and Animal Sciences, University of Milan (UNIMI), 26900 Lodi, Italy; 3 Institute of Animal Welfare Science, University of Veterinary Medicine Vienna, Veterinaerplatz 1, 1210 Vienna, Austria; 4 MTA-ELTE Lendület “Momentum” Companion Animal Research Group, Budapest, Hungary

**Keywords:** Cognitive ageing, Social behaviour, Animal behaviour

## Abstract

The prolonged lifespan of companion dogs has resulted in increased behavioural and physical challenges linked to old age. The development of behavioural tests to identify and monitor age-related differences has begun. However, standardised testing requires validation. The present study aimed to assess external validity, interobserver reliability, and test–retest reliability of an indoor test battery for the rapid assessment of age-related behavioural differences in dogs. Two experimenters tested young dogs (N = 20, mean age ± SD = 2.7 ± 0.4 years) and old dogs (N = 18, mean age ± SD = 11.8 ± 1.3 years) in the test battery once and then again after two weeks. Our results found external validity for two subtests out of six. On both test occasions, old dogs committed more errors than young dogs in a memory subtest and showed more object avoidance when encountering a novel object. Interobserver reliability and test–retest reliability was high. We conclude that the Memory and Novel object subtests are valid and reliable for monitoring age-related memory performance and object neophobic differences in dogs.

## Introduction

The lifespan of pet or companion dogs has been increasing over the years^1^. Consequently, behavioural and physical deficits in old age have become more prevalent. In the last decades, research on canine ageing has grown exponentially as both scientists and the public have increasingly recognised dogs’ emotional, economic, and scientific value as an animal model species^2–5^. Studies have shown that owners of ageing pet dogs often report a decline in the dogs’ visual and auditory function, changes in social behaviour^6–12^, and the sleep/wake cycle^13,14^.

To better understand these phenomena, researchers have developed various behavioural tests to measure the behavioural differences that occur with old age in companion dogs. For instance, a curiosity test showed that the chronological age of the dogs is linked to their neophilic behaviour: specifically, young dogs (1–4 years) sniffed and played for a longer time with novel objects compared to older dogs (> 9 years)^15^. In a similar study, in the presence of an unfamiliar person, younger dogs (1 4 years) physically interacted more frequently with them compared to older dogs (> 9 years)^16^.

Previous studies also demonstrated an impairment of several cognitive abilities such as memory, learning and flexibility in aged dogs^3,4,11,15^. For instance, Piotti and colleagues^4^ showed that, in discrimination and reversal learning tasks, younger dogs (1.5–6.5 years) were able to learn faster than older dogs (8.0–14.5 years). These results have been further validated using EEG, demonstrating a correlation between sleep spindle (non-REM bursts of activity in the sigma range) intrinsic frequency and the number of reversal learning training trials required to reach the criterion^17^. Sleep spindles predict learning in dogs and vary with age^18,19^. Ageing appeared to affect also dogs’ ability to retain and later exploit spatial information. Using a spatial memory task that required the use of short-term memory to find food, it has been found that younger dogs (3–6 years) were more efficient than older dogs (9–11 years), committing fewer errors and finding the food more often at their first attempt^3^. The relationship between the performance in the spatial memory task and the dogs’ gut microbiome was also investigated, suggesting a worse memory performance (more errors) was associated with a higher proportion of Actinobacteria in their faeces^20^. These findings are in agreement with the high abundance of some Actinobacteria found in the gastrointestinal tract of patients with Alzheimer’s disease^21^.

Recently a battery of standardised outdoor behavioural tests (Mini Mental Test, MMT) was developed to allow the rapid assessment of age-related behavioural differences in family dogs^2^. Older dogs displayed less social interest, poorer spatial memory, and seemed less interested in and less fearful of a novel, moving object^2^. However, neither test–retest nor interobserver reliabilities were reported for this test battery which are necessary before applying the tests to clinical settings.

The development and quality of behavioural assessments should be assessed through five key measures: defining the test’s purpose, standardisation, reliability, validity, and practicality (or feasibility)^22^. A biological measurement is the cumulative result of several factors: the true value of the phenomenon that we intend to measure, biological variation, tool sensitivity, the skill and expectation of the observer and the experimenter, subject-related factors (e.g., hunger, fear), as well as external factors, such as environmental temperature or visual, olfactory, and auditory stimuli^23^. In a standardised test, two parameters are assessed to measure if the test can be considered relevant and accurate, reliability and validity^24,25^.

A measure is considered reliable when it is consistent and stable over multiple measurements^25^. There are three criteria for reliability: 1) intra- and interobserver reliability or agreement, which is the level of consistency within and between observers/coders, assessing the effect of subjective bias on the coding/scoring system^25^; 2) internal consistency, indicating coherence among components of a scale aimed to measure the same phenomenon^26^; and 3) test–retest reliability, which shows that the test yields the same results when repeated on the same subjects under identical conditions^22^.

Validity indicates that the method measures what it is meant to measure, both internally and externally^22,26^. Internal validity relates to the value of the measure itself, and it is assessed through three categories^27^. Content validity or a test’s scientific relevance indicates that the method only contains measures relevant to its aims. Construct validity shows whether the hypothesised cause explains the test scores. Criterion validity (predictability) indicates the predictive ability of the measurement in comparison with a previously validated instrument (a “Gold Standard”). Finally, external validity is the degree to which results can be generalised across studies^27^.

Behavioural tests are frequently used in various contexts, for example, to assess temperament or personality in pet, working, and shelter dogs^22,27,28^, which may be assessed in person or remotely^26^.

Despite the widespread use and the importance of behavioural testing for ageing research, some shortcomings have been identified. Some tests require a long training interval and, therefore, cannot be repeated over a short period (e.g. ^4^,), which makes it impossible to use them to monitor age-related behaviour changes in a longitudinal study design^29^. Others rely on social interaction^16,30^, which different dogs may perceive differently depending on the partner. For example, test accuracy may be undermined by the different responsiveness of dogs towards male and female experimenters. Previous research indicates that shelter dogs show a stronger decrease in defensively-aggressive behaviours (tendency to look, bark) towards women^31^, lower levels of plasma cortisol and more relaxed posture when petted by women^32^, as well as more stress-related behaviours (tendency to look, shorter tail-high periods, lip-licking) when walked on a leash by men^33^. The influence of human gender on behaviour has been understudied in companion dogs^34,35^ and, so far, has not been listed as a potential confounding factor in field tests aimed at assessing age-related interspecific social behavioural differences. Finally, cognitive tests designed to measure positive affective states have replicability issues and may not be reliable in ageing dogs due to the extensive learning required: for example, studies based on the cognitive bias test, a test for mood utilising discrimination choices, showed that older dogs might struggle to learn the discrimination and therefore it may not be possible to test them^4,36^. Currently, there are no standardised tests that can measure positive emotions in senior animals. Clinicians still need standardised testing for positive emotions in senior animals.

Previously we determined that the MMT demonstrated content and construct validity (internal validity) and a good degree of external validity^2,20^. This study aimed to investigate the reliability (interobserver, interexperimenter, test–retest) and reiterate the study of the internal validation (content and construct validity) of the MMT^2^, and adapt it to indoor settings to have a controlled environment with limited distractions. To measure interobserver and intra-experimenter agreement as well as test–retest reliability, we modified the protocol to include two experimenters (a woman and a man) and tested both old and young dogs and compared the dogs’ behaviour in the two situations (first occasion (T0), second after one to two weeks (T1)) with the different experimenters. The experimenters and an independent observer coded the dogs’ behaviour to calculate interobserver reliability. We also added a new test to the battery to assess spatial memory and neophilia, the Novel object recognition test (NOR)^37–39^. This test is widely used with murine models but, to our knowledge, has not been applied to dogs^37^.

## Methods

### Ethics

**Figure 1:**
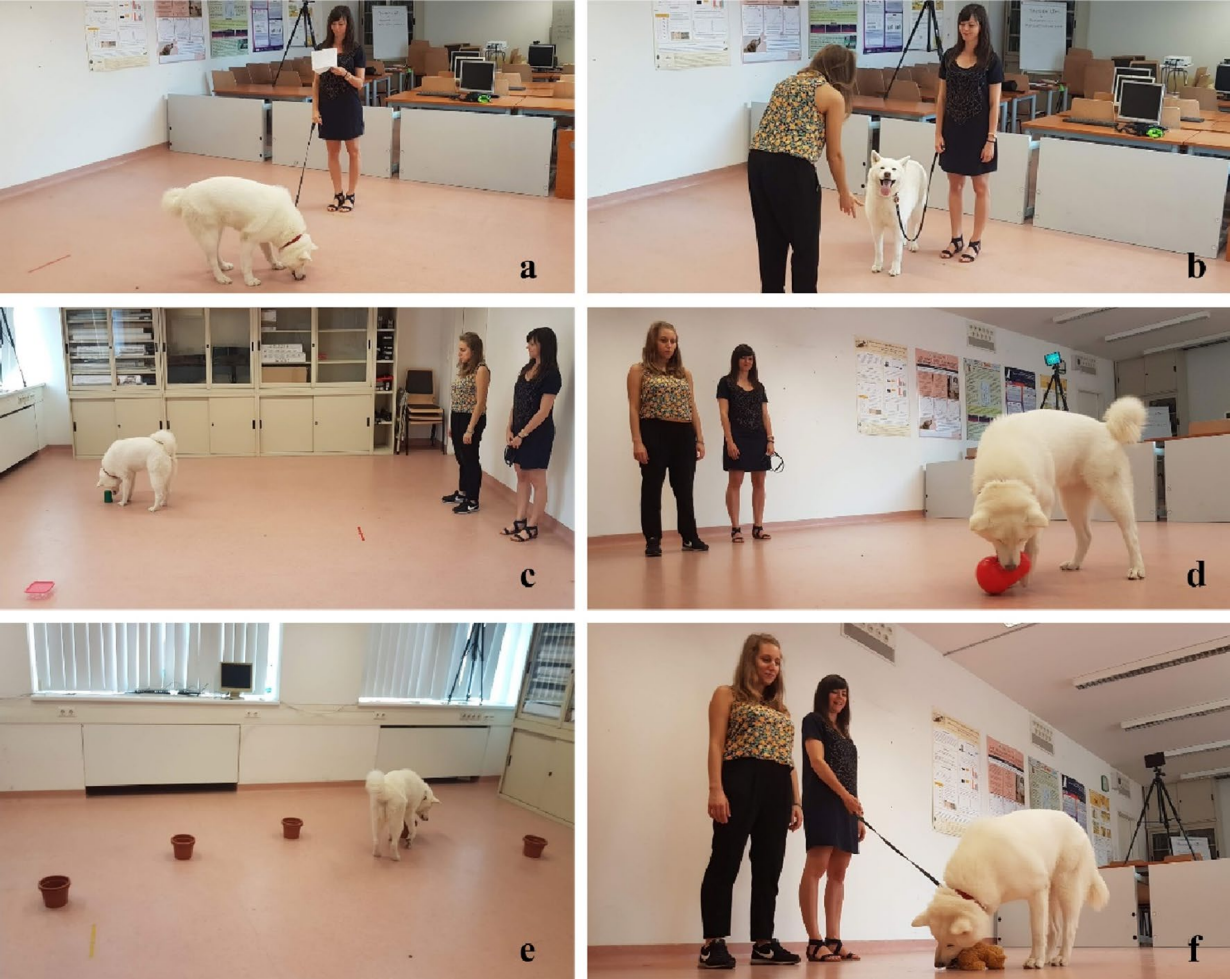
Behavioural tests of the test battery. (**a**) Exploration; (**b**) Greeting; (**c**) Novel object recognition; (**d**) Problem box; (**e**) Memory; (**f**) Novel object (toy dog).

All procedures complied with national and EU legislation and institutional guidelines in strict accordance with the International Society for Applied Ethology guidelines for the use of animals in research. The study received ethical permission from the Hungarian Pest County Governmental Office following the ethical review of the Eötvös Loránd University (Permission No.: PE/EA/2019–5/2017). Owners provided written consent for their voluntary participation. We took special care to ensure that the dog owners understood the consent process completely. In the consent form, participants were informed about the identity of the researchers, the procedure, location, expected time commitment of the experiment, handling of personal and research data, and data reuse. The owners were not informed about the exact aim of the tests. The information in the consent form included the participant’s right to withdraw their consent at any time. Participants could decline to participate at any point and request that their data not be used and/or deleted after they were collected. Our consent form was based on the Ethical Codex of Hungarian Psychologists (2004). For Fig. 1, we obtained informed consent from all subjects and/or their legal guardian(s) for publication of identifying information/images in an online open-access publication. For experiments involving human participants, written informed consent was obtained from all subjects and/or their legal guardian(s).

### Subjects

Thirty-eight dogs were recruited through the Department of Ethology, ELTE’s database of participants, social media, and word of mouth. Two groups of dogs were formed based on their age: ‘young dogs’ (N = 20, mean age ± SD = 2.7 ± 0.4, median age 3 years, IQR = 2.50–4.00, 50% female, 65% neutered), and ’old dogs’ (N = 18, mean age ± SD = 11.8 ± 1.3, median age 11 years, IQR = 10.62–12.88, 33% female, 78% neutered). Age categories (1–4 years for young dogs and above 9 years for old dogs) were based on previous findings regarding the onset of cognitive decline (see^40,41^ for a review). The sample included 14 mix-breeds and 24 pure breeds from 16 different breeds (Young: 6 mixed breeds, 3 golden retrievers, American Staffordshire terrier, Akita inu, Australian shepherd, Belgian shepherd, border collie, German shepherd dog, Hungarian sighthound, Kerry blue terrier, rottweiler, Siberian husky, standard poodle; Old: 8 mixed breeds, 2 border collies, American Staffordshire terrier, Belgian shepherd, golden retriever, Hungarian sighthound, labrador retriever, shar pei, vizsla, whippet; see Table S1 for full demographic information). The dogs were free from overt signs of distress and/or pain for both groups during the test.

### Procedure

The study was performed in an experimental room at the Department of Ethology, ELTE. Two tablets (Samsung Galaxy Tab S2), positioned at opposite corners of the room, recorded the behavioural performances of the dog during the test (Fig. 1).

The battery consisted of six indoor subtests (Fig. 1). An experimenter was present in the room for all subtests apart from the exploration test. The owner stood on his/her left-hand side and a coder, who coded some of the tests live, on his/her right-hand side. The owner kept the dog on the leash unless instructed differently.

The dogs underwent the same test twice (T0 = first test, T1 = second test, after 1 to 2 weeks) to measure test–retest reliability. Different objects were used in the second test when the dogs had to be naïve to a specific object (see Supplementary Material). The same experimenter and coder performed the test on both occasions. Half of the dogs were tested by a male experimenter the other half were tested by a female experimenter. The allocation to each experimenter was counterbalanced across dogs within age groups (see Table S2).

The behavioural variables measured are presented in Table 1.

**Table 1:** Subtests of the battery, variables, and their definition (modified from Kubinyi and Iotchev, 2020).

Subtest	Variable name	Type of variable	Definition
**Exploration**	Activity level	Ordinal	All four of the dog’s paws were moving and/or the dog’s nose was close to the ground (sniffing) Score: 1—the dog was active less than 10% of the time 2—the dog was active between 10 and 50% of the time 3—the dog was active between 50 and 90% of the time 4—the dog was active over 90% of the time
**Greeting**	Social interaction	Ordinal	Greeting behaviour towards the experimenter Score: 1—the dog avoided the experimenter and did not get close 2—the dog looked at the experimenter but did not get close 3—the dog moved closer to the experimenter after 5 s 4—the dog moved closer to the experimenter within 5 s
**Novel object recognition (NOR)**	Recognition Index (RI)	Continuous	Time spent investigating the novel object relative to the total object investigation time^37^
	Neophilic Behavior	Binomial (yes/no)	Indicates whether the dog explored the novel object before the familiar one when presented with the new pair
**Problem box**	Object manipulation	Ordinal	The dog manipulated the Kong while being within a head’s space of it and/or the dog touched it with their head or paw Score: 1—the dog did not touch the Kong 2—the dog touched the Kong but there was no manipulation 3—the dog manipulated the Kong for less than 50% of the time 4—the dog manipulated the Kong for more than 50% of the time
**Memory**	Errors	Frequency	Number of incorrect containers visited by the dog on each trial
	Spatial memory	Binomial (yes/no)	Whether the dog found the baited container on their first attempt or not
	Control Trials	Binomial (yes/no)	Whether the dog found the baited container on their first attempt or not
**Novel object (toy dog)**	Object interaction	Continuous	Proportion of time the dog spent interacting with the toy
	Object avoidance	Continuous	Proportion of time the dog spent moving away from the toy

#### Exploration

The goal of this subtest was to measure the dogs’ activity level and interest in investigating a novel environment^2,42,43^. The owner walked into the room with the dog on the leash and stayed in a pre-determined position (Fig. 1a) for one minute while reading a paper given by the experimenter (to prevent the owner from looking at or talking to their dog).

#### Greeting

This subtest aimed to measure the sociability of dogs toward unfamiliar friendly people^2,42,43^. The experimenter entered the room and greeted the dog (Fig. 1b). If the dog approached the experimenter, the interaction continued in a standardised way (see Supplemental Material), including a ball or tugging game.

#### Novel object recognition (NOR)

The goal of this subtest was to measure neophilia behaviour^44^ and short-term memory. Dogs were presented, in a pre-determined order (Table S2), with two pairs of containers with different shapes and colours (Fig. S1). After one minute of exploring them, the dog was taken out of the room (Fig. 1c). The experimenter swapped the containers with a new pair, where one container was identical to the first one, and the second container had a novel shape and colour. The dog-owner dyad re-entered the room, and the dog had one minute to explore the containers. The position of the novel container and the types of containers were pseudo-randomised and counterbalanced between dogs and between T0 and T1 (Table S2).

#### Problem box

This subtest aimed to measure the dogs’ persistence. The dog was presented with a food toy (Kong wobbler (Fig. 1d)), filled with 20 pieces of dry food, and had one minute to try and retrieve the food by manipulating the toy with the paw or mouth to make the food drop from a small hole (‘solvable task’). Then the experimenter filled the toy again with a single large piece of dry meat, which was too big to get through the hole, so it was not possible for the dog to retrieve the food (‘unsolvable task’). The dog was given the toy for one minute. None of the dogs had previous experience with this type of toy.

#### Memory

The goal of this subtest was to detect differences in the dogs’ short-term spatial memory. The dogs were presented with five identical containers (Fig. S2) placed in a semi-circle (Fig. 1e). The experimenter placed a piece of food in one of the containers, which the dog was allowed to retrieve after a break outside the room, according to the procedure described in Piotti et al. ^3^. The procedure was repeated five times, once per container, and the order of the baited container’s location was counterbalanced and pseudo-randomised across participants and varied between T0 and T1 (Table S2). In addition, at the end of T1, the dogs were presented with three additional trials (‘Control Trials’) where the location of the baited container was changed while the dog was prevented from seeing the baiting. This was done to exclude the possibility that the dogs followed odour cues in this subtest.

#### Novel object (toy dog)

The objective of this test was to measure dogs’ neophilia and neophobia. The dogs were presented for 30 s with an electronic, moving toy dog (Fig. 1f) placed on the ground by the experimenter, according to the procedure described in Kubinyi and Iotchev^2^. Two toys, identical in shape and rough movement, but different in colours and sound, were used at T0 and T1, and the order was counterbalanced across dogs (Fig. S1 and Table S2).

### Statistical analysis

Analyses were performed using R statistical software^45^ and the packages psych^46^, ordinal^47^, and lme4^48^. Cumulative linked mixed models (CLMMs) were calculated to analyse ordinal (score) data. The cauchit link function was used for the activity level variable, probit link function for the social interaction variable, and LogLog link function for the object manipulation variable.

Generalised linear mixed models (GLMMs) were used to analyse frequency, continuous and binomial data. The recognition index, memory errors, object avoidance, and object interaction variables had Poisson error distribution, while the neophilic behaviour and spatial memory/control trials had binomial error distribution.

For each model, we initially created a global model including all the variables of interest as fixed factors, with no interactions, and the dog as a random factor. Each global model included ‘age group’ (old vs young), ‘test number’ (test vs retest), and ‘experimenter’ (A vs. B) as fixed factors. The model for the predictor’ object manipulation’ also included the variable’ test phase’ (solvable vs unsolvable). The global models for the predictors’ errors’ and ‘spatial memory’ included ‘trial’ (1 to 5). The main factors’ age group’, ‘test number’ and ‘experimenter’ were maintained in all models as part of our main hypothesis, while for all other factors, we adopted a stepwise approach to select the most parsimonious model to describe the variance of each response variable. Pairwise post-hoc comparisons with Tukey correction were then obtained.

We used a Wilcoxon signed rank test to compare the proportion of times the dogs chose the baited container in trials 1, 3, and 5 at T1 with the proportion during the corresponding control trials in the Memory test.

Finally, an independent coder (AS) coded 20% of the video material (16 tests out of 76, from both T0 and T1), and interobserver reliability was assessed using interobserver agreement (kappa) for scores, Cronbach’s alpha for binary data, and Spearman correlations for count data.

### Ethical approval

The procedures of this study complied with national and EU legislation and institutional guidelines. The study received Ethical Permission from the Hungarian Pest County Governmental Office following the ethical review of the Eötvös Loránd University (Permission No.: PE/EA/2019–5/2017).

## Results

### Age group

**Figure 2:**
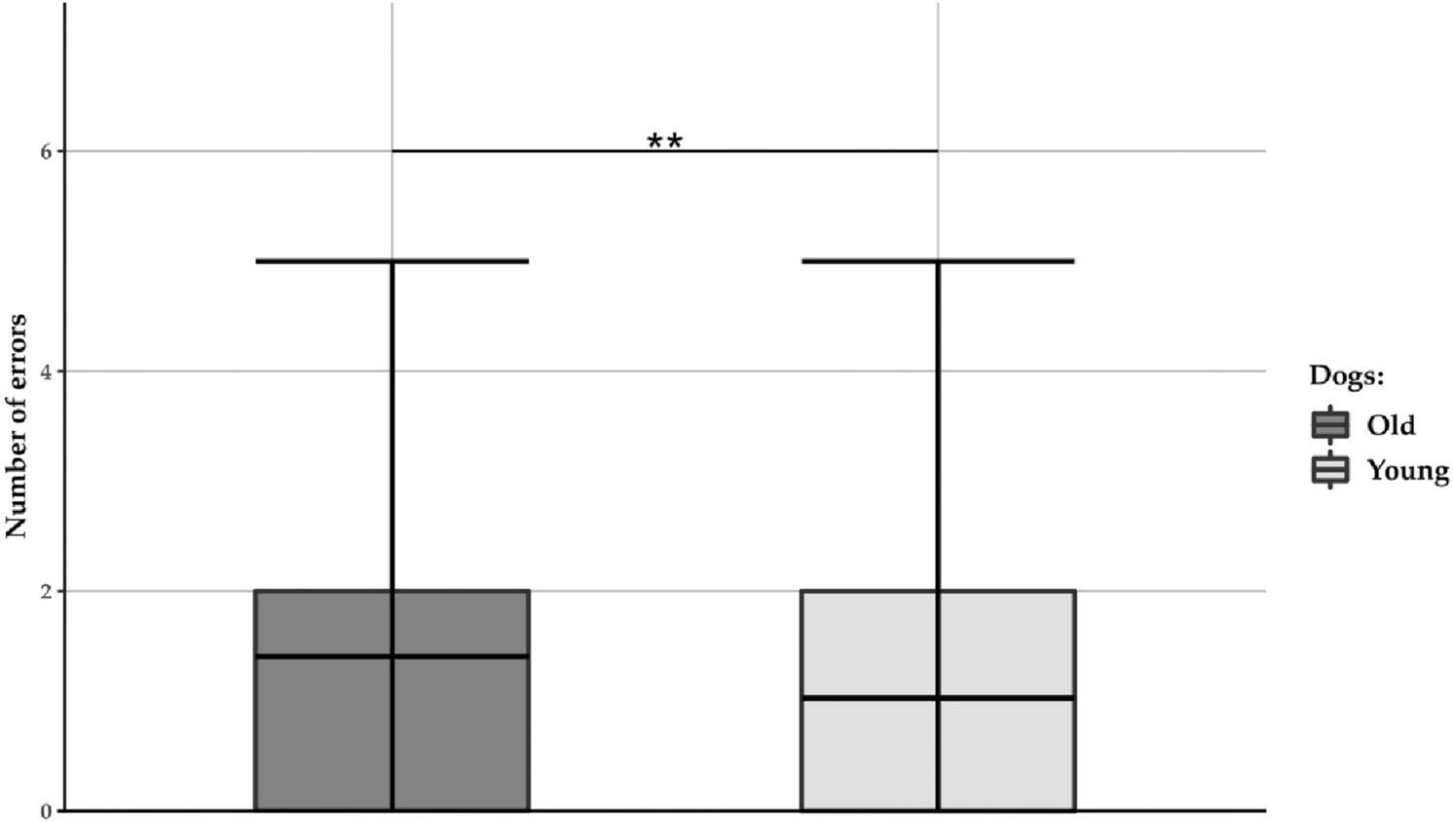
Number of errors in the memory test. On average, the old dogs made more errors in the memory test compared to the young dogs. A breakdown of the number of errors made in the memory test, divided by age group, is presented in the figure. The middle line in the box plots represents the median number of errors, the extremes of the boxes represent the lower and upper quartiles, and the error bars represent the minimum and the maximum number of errors. The asterisks indicate a statistically significant difference between the groups (** = *p* < 0.01).

**Figure 3:**
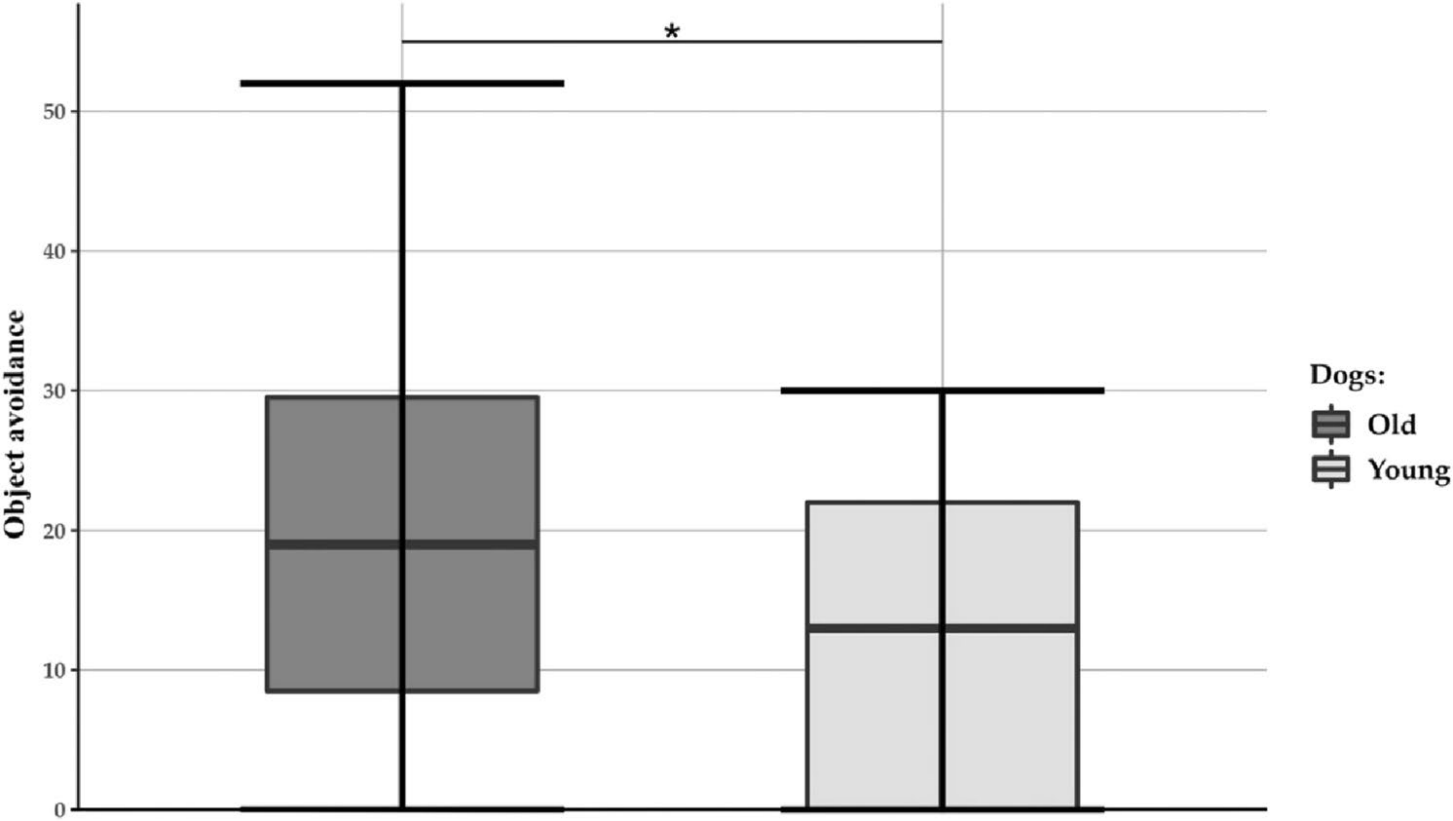
Object avoidance in the Novel object (toy dog) test. The old dogs avoided the toy for a larger proportion of time compared to the young dogs. A breakdown of the percentage of time spent avoiding the toy, divided by age group, is presented in the figure. The middle line in the box plots represents the median proportion of time spent avoiding the toy, the extremes of the boxes represent the lower and upper quartiles, and the error bars represent the minimum and maximum proportion of the time. The asterisk indicates a statistically significant difference between the groups (* = *p* < 0.05).

Young and old dogs differed in four variables from three subtests (Table 2). Young dogs were more likely to interact with the novel object first during the NOR, they chose fewer incorrect containers in the Memory subtest (Fig. 2, Fig. S3), and they interacted for a longer time with the toy dog and showed shorter avoidance behaviour in the Novel object (toy dog) subtest (Fig. 3, Fig. S4).

**Table 2:** The results of three cumulative linked mixed models (CLMMs) and six generalised linear mixed models (GLMMs). For each predictor, the estimate, the standard error (S.E. in brackets), and the p value (in italics) are reported. Significant p values are bolded.

CLMMs					
Test	Variable	Predictors			
		Age group: Young vs Old	Test–retest: T0 vs. T1	Experimenter: Male vs Female	Test Phase: Solvable vs. Unsolvable
**Exploration**	**Activity level**	− 0.19 (0.30) *0.529*	0.47 (0.26) *0.075*	0.46 (0.31) *0.133*	–
**Greeting**	**Social interaction**	0.08 (0.72) *0.902*	− 1.02 (0.45) ***0.023***	1.26 (0.91) 0.163	–
**Problem box**	**Object manipulation**	0.65 (0.55) *0.233*	0.37 (0.20) *0.062*	1.06 (0.56) *0.057*	0.63 (0.20) ***0.002***

GLMMs					
Test	Variables	Predictors			
		Age group: Young vs. Old	Test–retest: T0 vs. T1	Experimenter: Male vs. Female	Trial
**Novel object recognition (NOR)**	**Recognition Index**^†^	− 0.28^a^ (0.24) *0.138*	− 1.30^a^ < 0.001 (0.05) ***< 0.001***	1.13^a^ (0.95) *0.883*	–
	**Neophilic behaviour**	− 0.20^b^ (0.14) ***0.025***	− 6.53^b^ (4.57) ***0.007***	1.16^b^ (0.73) *0.810*	–
**Memory**	**Errors**	− 1.43^a^ (0.17) ***0.004***	0.95^a^ (0.09) *0.617*	0.97^a^ (0.12) *0.817*	–
	**Spatial memory**	0.84^a^ (1.04) *0.894*	8.93^a^ (7.56) ***0.009***	1.27^a^ (1.55) *0.845*	0.25 (0.23) 0.279
**Novel object (toy dog)**	**Object interaction**	0.43^a^ (0.13) ***0.005***	0.83^a^ (0.03) ***< 0.001***	1.11^a^ (0.33) *0.735*	–
	**Object avoidance**	− 2.47^a^ (1.06) ***0.035***	1.05^a^ (0.06) *0.449*	1.19^a^ (0.50) *0.683*	–

^a^estimate is given as ratio

^b^estimate is given as odds ratio

^†^*N* = 1 dog (Irisz) was excluded due to a technical issue with the camera

The results for the factor’ trial’ are not reported in the model for the variable ‘errors’ because this factor was not retained in the most parsimonious model.

### Test–retest

The dogs’ behaviour differed in four variables of three subtests. On the second test occasion, social interaction scores were higher than in T0 in the Greeting subtest, i.e. the dogs were quicker to move closer to the experimenter. The Recognition Index and the neophilic behaviour were lower compared to T0 in the NOR subtest, indicating that the dogs spent less time investigating the novel object as they were less interested in it. Finally, the spatial memory scores were lower in the Memory test, i.e. the dogs found the baited container less frequently during the second test occasion (Table 2).

### Experimenter

There were no significant differences between dogs tested by the male and the female experimenter in any of the variables measured (all *p* > 0.05; Table 2).

### Control trials in the memory subtest

The dogs were more successful in choosing the baited container when it was in the location they had witnessed during T1 (Trial 1: *p* = 0.008; Trial 3: *p* < 0.001; Trial 5: *p* = 0.003), compared to the control trials (see Table S3 for statistical details).

### Interobserver agreement

Interobserver agreement (kappa), Chronbach’s alpha, and Spearman correlations indicated excellent agreement between coders as all values were equal to 1 and all *p* < 0.001 (Table S4).

## Discussion

The first goal of this study was to measure the reliability of a battery of six indoor behavioural subtests for the rapid assessment of behavioural differences between young and old companion dogs. Our results indicate that the variables object avoidance in the Novel object (toy dog) subtest and the errors in the Memory subtest are reliable and can be used to monitor age-related behavioural changes in companion dogs. Both measures were unaffected by the experimenter’s identity or the re-testing. Furthermore, these variables were associated with good interobserver reliability (see Supplementary Material), confirming that the subtest coding was well standardised.

The second aim was to reproduce the results obtained in our previous studies^2,3^ in an indoor setting. The Novel object (toy dog) subtest confirmed the large effect of age previously observed^2^. Younger dogs were much less avoidant of the toy than older animals, meaning that the time they spent moving away from the toy was shorter.

Avoidance is a behavioural manifestation of fear or anxiety^49^, which is known to increase in dogs as they age^13^. Age-related changes in the regulation of emotions in dogs are thought to depend on the degeneration of the amygdala, causing increased sensitivity to positive stimuli^7^. However, anxiety in senior dogs may be caused by multiple reasons, such as central or peripheric neuropathology, sensory decline^6^, metabolic, gastrointestinal or urogenital disease, dermatological conditions, pain^13^, or underlying behaviour problems which aggravate with time^50–52^.

During the Novel object subtest, younger dogs spent more time interacting with the toy dog than older dogs. Persistence in interaction with objects might depend on differences in motivation^53^, which may decline in ageing dogs due to cognitive or physical changes. Moreover, some dogs may have perceived their interaction with the object as a playing activity, and the results could indicate a stronger inclination for playfulness in younger individuals. Playing is a sign of positive emotional states^54–56^, which are fundamental for the individual’s quality of life and should therefore be monitored in senior dogs^57^. Nevertheless, despite a significant difference between young and old dogs, according to our results, the variable Object interaction in the Novel object subtest displays a re-test effect; therefore, this variable should not be coded over time.

We also replicated our previous findings of a reduced short-term spatial memory performance in aged dogs compared to young dogs^2,3^, thus confirming the efficiency of this subtest in detecting age-related differences in an independent population of companion dogs. Older dogs more often chose the wrong locations compared to younger dogs. The control trials excluded the possibility that the dogs followed odour cues during the subtest. Therefore, we can conclude that they relied on the visual information they had gathered during the first part of the subtest to find the hidden food in the second part. These findings indicate that the Memory subtest is a reliable and valid behavioural test which could be used to monitor longitudinal changes in dogs’ spatial memory.

Previously, we demonstrated that the Memory subtest has a correlation between errors in the Memory subtest and the canine gut microbiome composition was observed^20^. This finding will have a large practical impact on the welfare of dogs, as it will allow veterinarians and other animal professionals to perform a standardised, reliable, valid, practical test to monitor an important cognitive skill as the dogs age. Such tests are fundamental for distinguishing between normal and pathological ageing^41^, as well as for monitoring the progress of age-related pathologies, such as Cognitive Dysfunction Syndrome^58^. The cognitive decline caused by other medical conditions, such as epilepsy^59^, could also be monitored.

Furthermore, for all the other subtests, we did not reproduce the previous findings. Kubinyi and Iotchev^2^ detected a small age effect in the problem box subtest in an outdoor setting, but the present study suggests that, even if the test appears to be consistent over time, it seems to have no construct validity for ageing itself. Similar findings were observed in the study by González-Martinez et al.^60^, where the authors found significant differences between groups at different levels of cognitive decline (young dogs vs. aged dogs at normal cognitive levels and aged dogs with impaired cognitive levels); however, the authors did not detect significant differences based on age groups (1–4 years, 5–8 years, 9 years and above). In the current study, young and old dogs manipulated the object similarly. Thus, the test may not be consistently effective in detecting age-related behavioural modifications in companion dogs. Similarly, during the exploration and greeting, we did not detect a significant difference in social interaction between young and old dogs, and this variable seems to be affected by the repetition of the test. Therefore, this subtest should not be considered reliable and suitable for longitudinal evaluations of ageing, at least not in indoor tests. Activity levels in the Exploration subtest were consistent between T0 and T1, but old and young dogs’ performances did not vary significantly, meaning that the dogs’ exploratory behaviour in this subtest was not an effective measure of ageing. According to these findings, the Exploration and Greeting tasks should not be employed to monitor age-related differences in companion dogs as described in the current protocol.

This battery of subtests presented for the first time a paradigm to measure novel object recognition (NOR) in family dogs. Contrary to what is largely observed in other species, such as murine models^37–39^, we did not find a difference between younger and older dogs in the standard measure of the recognition of the novel object, the Recognition Index. However, the older dogs demonstrated lower neophilic behaviour (i.e., fewer dogs explored the novel object first) than younger dogs. While this difference may not be predictive of cognitive decline, it is in line with the findings on object interaction in the Novel object toy dog subtest, suggesting a decrease in curiosity and motivation towards objects with age.

Although some of the subtests of the battery have detected reliable behavioural differences associated with ageing, we must point out that the present results are restricted to a specific population of companion dogs. Firstly, the subjects tested in this study did not suffer from any overt medical conditions, and the dogs did not undergo a cognitive assessment. Therefore, the present results reflect age-related behavioural differences associated with ageing. Further investigations may help evaluate how and to what extent certain pathologies could influence senior dogs’ behavioural performances in the subtests, which could result in the development of assessment tools to aid in the diagnosis of medical conditions, including Canine Cognitive Dysfunction. Secondly, both groups of dogs were medium to large-sized. It is well known that the ageing process is strongly associated with body size, as small-sized dogs age more slowly than large-sized dogs^41^. However, it is unclear whether the subject’s size is a confounding variable in subtests that assess age-related behavioural differences in dogs. Since dogs’ body size has not yet been taken into account, future studies should also evaluate the potential effect of this factor to ensure high external validity.

## Conclusion

It is often complicated to clinically separate medical and behavioural conditions in senior dogs^13,52^. The presence of pathologies, such as cognitive impairment, is usually related to a modification of behaviours (i.e. disorientation, altered interactions, anxiety) and is often difficult to quantify for both the owners and clinicians^13,52^. Moreover, factors such as breed and individual differences may further confound the correlation between behavioural modifications and specific clinical conditions^13^. For these reasons, standardised behavioural tests are particularly useful as they may aid the diagnosis and monitoring of age-related changes in dogs, allowing us to make a more apparent distinction between healthy and pathological ageing processes.

Overall, the current findings indicate that two tests with two variables are suitable for assessing age-related differences in companion dogs, namely the ‘errors’ in the Memory test and ‘object avoidance’ in the Novel object (toy dog) test. These variables have good interobserver and inter-experimenter agreement, as well as test–retest reliability. Taking into account previous research, too^2,20^, the Memory test is both valid and reliable. The Novel object (toy dog) test also appears reliable and demonstrates good external validity; further studies should investigate its internal validity. Since these tests are consistent over time, they can be used for monitoring age-related changes in dogs in longitudinal research and the relationship of the performance with medical conditions, including Canine Cognitive Dysfunction.

## Supplementary Information

Supplementary Information.

## Data Availability

Data are available upon request from the first author. Correspondence and requests for materials should be addressed to E.K.
