# Supplementary material for: Two valid and reliable tests for monitoring age-related memory performance and neophobia differences in dogs
Source: Sci Rep. 2022 Sep 28;12:16175. doi: 10.1038/s41598-022-19918-7 (PMC9519567; doi:10.1038/s41598-022-19918-7)
Supplement: Supplementary file 1 — Supplementary Information. [file 41598_2022_19918_MOESM1_ESM.pdf]

Supplementary material

Two valid and reliable tests for monitoring age-related memory performance and neophobia differences in dogs

Patrizia Piotti[1,2,#], Andrea Piseddu[1,3,#], Enrica Aguzzoli[1], Andrea Sommese[1] and Eniko Kubinyi[1,4,*]
[1]Department of Ethology, ELTE Eötvös Loránd University, Pázmány Péter sétány 1/c, 1117, Budapest, Hungary
[2]Department of Veterinary Medicine, University of Milan (UNIMI), 20133 Milan, Italy
[3]Institute of Animal Welfare Science, University of Veterinary Medicine Vienna, Veterinaerplatz 1, 1210 Vienna, Austria
[4]MTA-ELTE Lendület "Momentum" Companion Animal Research Group

[#]These authors contributed equally to this study
*Correspondence: eniko.kubinyi@ttk.elte.hu

Table S1. Demographic information of the dogs

Testing order	Dog name	Age (y)	Age Group	Sex	Neutered	Breed	Height (cm)	Weight (kg)	Experimenter	Coder
1	Mustár	11.0	Old	M	Y	Mix breed	40	20	AP	EA
2	Carlos	12.0	Old	M	N	Vizsla	65	28	EA	AP
3	Dió	11.0	Old	M	Y	Golden retriever	60	35	EA	AP
4	Cooper	13.0	Old	M	Y	Border collie	52	21	AP	EA
5	Polka	3.0	Young	F	Y	Border collie	51	15	AP	EA
6	Scooby	15.0	Old	M	Y	Border collie	51	23	AP	EA
7	Muffin	2.5	Young	F	N	Australian shepherd	50	19	EA	AP
8	Mackó	10.0	Old	M	Y	Labrador	60	34	AP	EA
9	Mini	11.0	Old	F	Y	Mix breed	46	17	AP	EA
10	Írisz	4.0	Young	F	N	Standard poodle	68	19	EA	AP
11	Maci	13.0	Old	M	Y	Mix breed	40	25	EA	AP
12	Prince	3.0	Young	M	N	German shepherd	72	37	AP	EA
13	Doró	2.0	Young	F	Y	Rottweiler	58	30	EA	AP
14	Kósza	2.0	Young	M	N	Belgian shepherd	60	20	EA	AP
15	Berci	12.0	Old	M	Y	Mix breed	42	21	EA	AP
16	Floyd	4.0	Young	M	N	Golden retriever	60	28	EA	AP
17	Bogyó	2.0	Young	M	Y	Mix breed	33	30	AP	EA
18	Rufi	4.0	Young	F	Y	Mix breed	48	14	EA	AP
19	Leila	2.0	Young	F	Y	Golden retriever	45	30	AP	EA
20	Lili	11.0	Old	F	N	Whippet	49	16	AP	EA
21	Bizsu	4.0	Young	F	Y	Siberian husky	63	23	EA	AP
22	Popeye	12.5	Old	M	Y	Shar Pei	55	20	AP	EA
23	Tala	11.0	Old	M	Y	Hungarian sighthound	57	30	EA	AP
24	Buch	10.5	Old	M	Y	Mix breed	25	30	AP	AS
25	Smafu	10.0	Old	F	Y	Mix breed	57	24	EA	AP
26	Szőrmi	2.5	Young	M	Y	Mix breed	46	25	AP	AS
27	Gomez	3.0	Young	M	Y	Mix breed	60	29	AP	EA
28	Lupi	10.0	Old	M	N	Mix breed	74	44	EA	AP
29	Mogyoró	4.0	Young	F	Y	Mix breed	39	10	EA	AP
30	DióE	2.5	Young	F	Y	Hungarian sighthound	75	31	AP	EA
31	Panda	13.5	Old	F	Y	American staffordshire terrier	50	25	EA	AP
32	Zafír	3.0	Young	M	N	Kerry blue terrier	49	15	EA	AS
33	Cheyenne	10.5	Old	F	N	Belgian shepherd	63	24	AP	AS
34	Szoli	13.0	Old	F	Y	Mix breed	57	29	EA	AP

35	Dingó	3.5	Young	M	N	American staffordshire terrier	47	25	AP	AS
36	Bogi	4.0	Young	F	Y	Akita inu	63	27	AP	EA
37	Mala	5.0	Young	M	Y	Golden retriever	59	43	AP	EA
38	Kócos	2.5	Young	M	Y	Mix breed	41	15	EA	AP

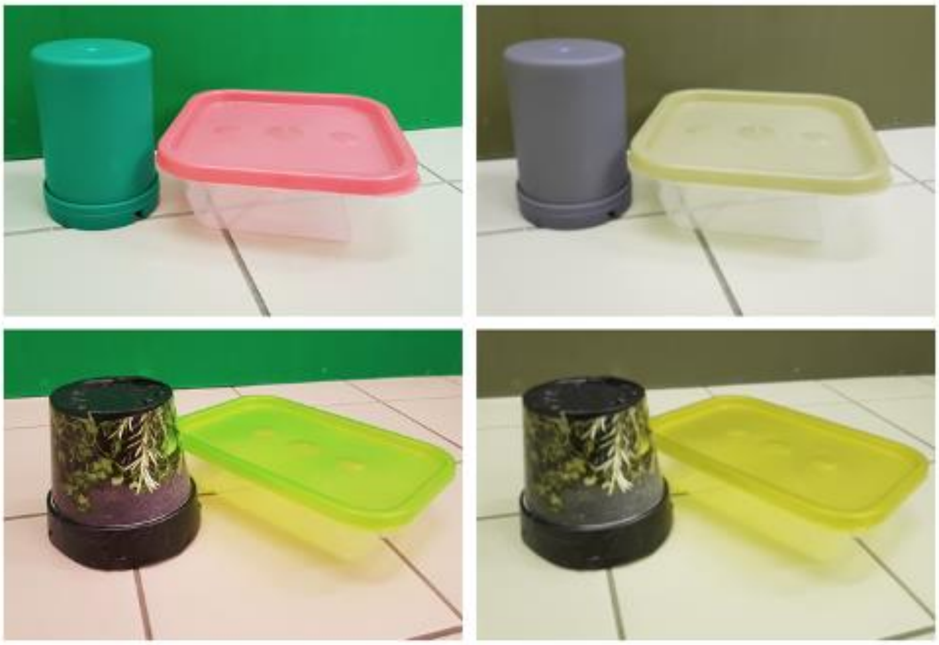

Figure S1. Objects used in the Novel Object Recognition test. A rectangular green Tupperware (‘Tupp. Green’), a squared pink Tupperware (‘Tupp. Pink’), a cylindric green container (‘Tube Green’), and a cylindric grey container (‘Tube Grey’).). To obtain contrast in shape and colour, pink squared containers were always presented with cylindric green containers, whereas rectangular green containers with cylindric grey containers. Dog perception of contrast in both colour and brightness of the combined containers was assessed using the website Dog VISION (https://dog-vision.andraspeter.com/). To avoid bias due to odour marks, the containers were previously cleaned using a solution of 70% (vol/vol) ethanol and were manipulated wearing latex gloves. The boxes’ combinations were counterbalanced between dogs and sessions (test, re-test).

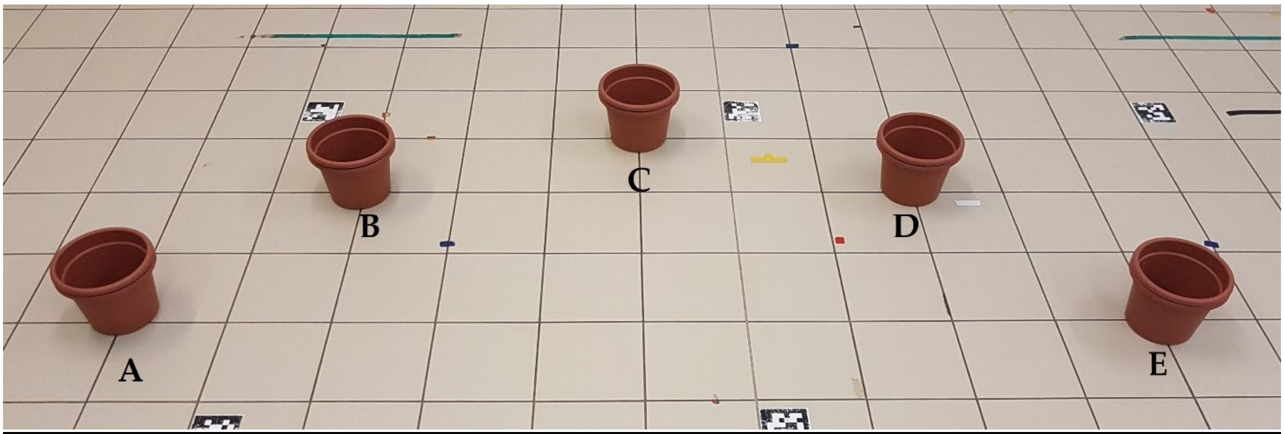

Figure S2. Randomisation and counterbalancing of the containers used for the Memory test.

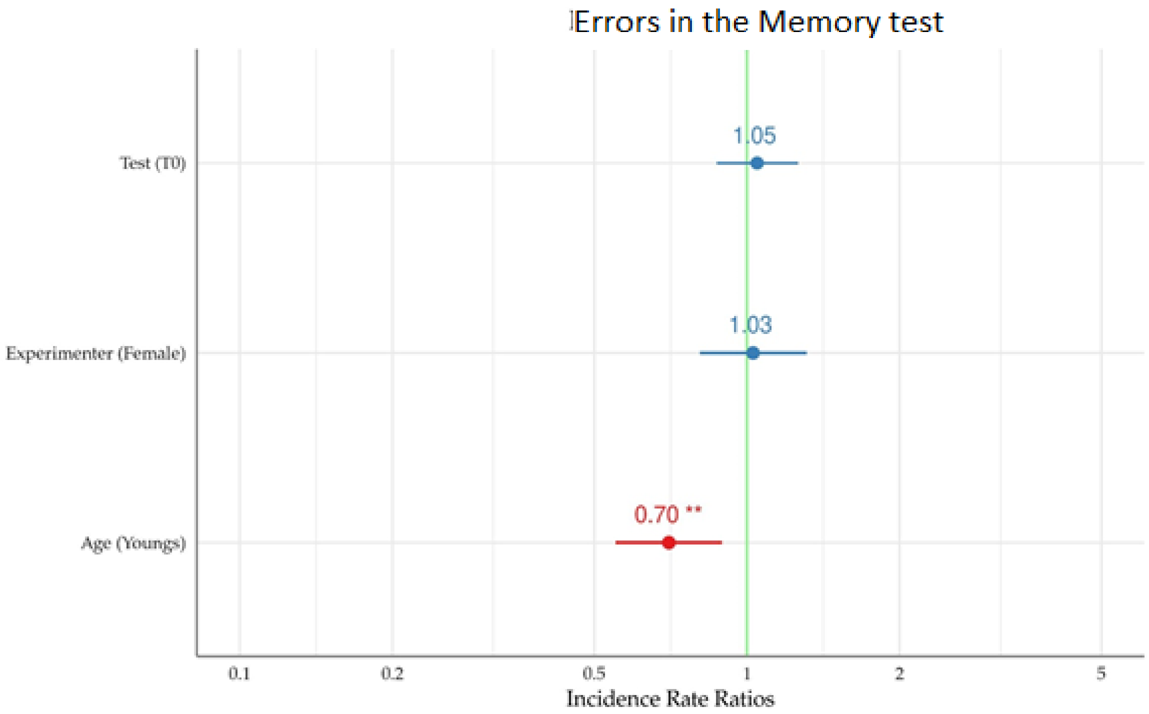

Figure S3. Forest-plot representing the incidence rate ratios of the errors for the fixed factors Age group (old, young), Experimenter (male, female), and Test Type (T0, T1) in the Memory test. The plot shows all the results of the generalised linear mixed model calculated to test the influence of the tree fixed factors on response variable mnemonic errors. The vertical intercept marked in green indicates no effect of the factors. The asterisks indicate a statistically significant difference between the groups (** = $p < 0.01$).

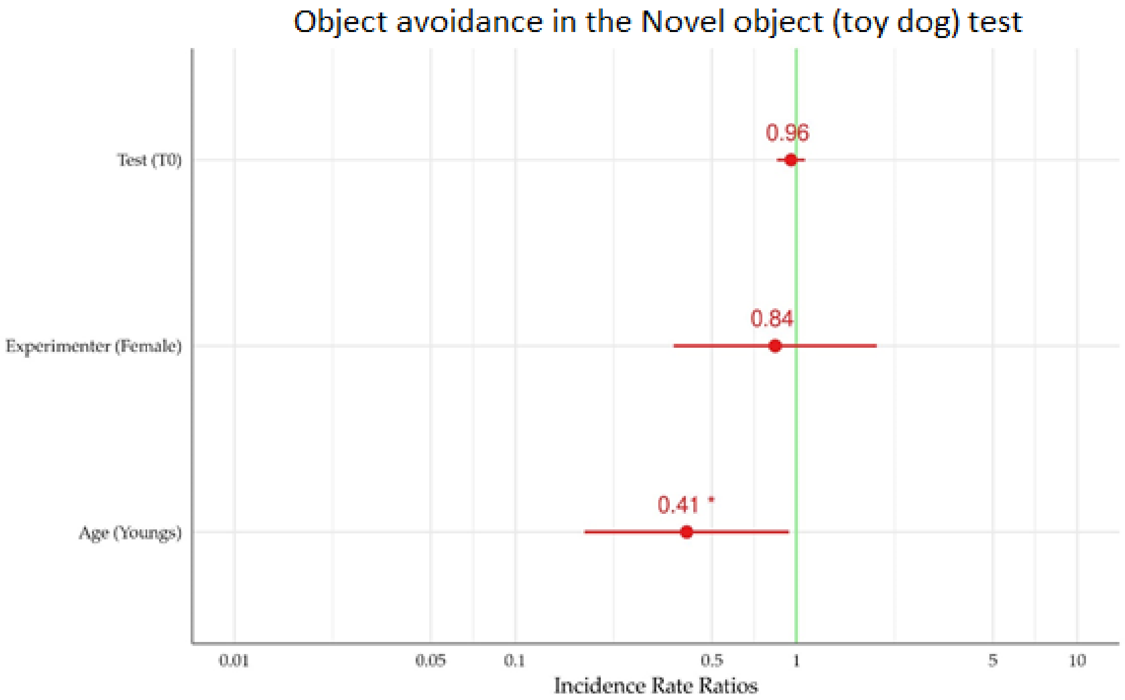

Figure S4. Forest-plot representing the incidence rate ratios of the object avoidance for the fixed factors Age group (old, young), Experimenter (male, female), and Test Type (T0, T1) in the Novel object (toy dog) test. The plot shows all the results of the generalised linear mixed model calculated to test the influence of the tree fixed factors on response variable object avoidance. The vertical intercept marked in green indicates no effect of the factors. The asterisk indicates a statistically significant difference between the groups (* = $p < 0.05$).

Table S2. Randomisation and Counterbalancing for the Novel Object Recognition (NOR) and Memory test in both testing occasions (T0 and T1). The dogs were allocated based on their age group and the order of recruitment. The first baited location (Memory test, see Figure S2) was counterbalanced across dogs, the following locations were predetermined randomly (www.random.org).

Dog	Age group	Experimenter	T_0 (NOR side)	T_0 (Memory baited locations' order)	T_0 (Objects NOR)	T_1 (NOR side)	T_1 (Memory baited locations' order)	T_1 (Objects NOR)
Muffin	Young	Female	Left	A, D, E, B, C	Tupp. Pink + Tube Green	Right	E, A, D, B, C	Tube Grey + Tupp. Green
Írisz	Young	Female	Left	B, C, A, E, D	Tupp. Pink + Tube Green	Right	D, C,B, E, A	Tube Grey + Tupp. Green
Kósza	Young	Female	Left	C, B, D, E, A	Tupp. Pink + Tube Green	Right	C, D, B, A, E	Tube Grey + Tupp. Green
Bogyó	Young	Male	Left	D, B, A, C, E	Tupp. Pink + Tube Green	Right	B, E, C, A, D	Tube Grey + Tupp. Green
Leila	Young	Male	Left	E, A, D, B, C	Tupp. Pink + Tube Green	Right	A, B, D, C, E	Tube Grey + Tupp. Green
Polka	Young	Male	Right	A, C, E, D, B	Tupp. Green + Tube Grey	Left	E, C, A, D, B	Tube Green + Tupp. Pink
Prince	Young	Male	Right	B, E, A, D, C	Tupp. Green + Tube Grey	Left	D, B, A, C, E	Tube Green + Tupp. Pink
Doró	Young	Female	Right	C, D, E, A, B	Tupp. Green + Tube Grey	Left	C, E, A, D, B	Tube Green + Tupp. Pink
Floyd	Young	Female	Right	D, E, A, B, C	Tupp. Green + Tube Grey	Left	B, C, E, D, A	Tube Green + Tupp. Pink
Rufi	Young	Female	Right	E, C, A, B, D	Tupp. Green + Tube Grey	Left	A, D, E, C, B	Tube Green + Tupp. Pink
Mustár	Old	Male	Left	A, D, B, C, E	Tupp. Pink + Tube green	Right	E, C, D, B, A	Tube Grey + Tupp. Green
Cooper	Old	Male	Left	B, C, E, A, D	Tupp. Pink + Tube Green	Right	D, C, A, E, B	Tube Grey + Tupp. Green
Mini	Old	Male	Left	C, E, A, D, B	Tupp. Pink + Tube Green	Right	C, E, D, A, B	Tube Grey Tupp Green
Maci	Old	Female	Left	D, E, A, B, C	Tupp. Pink + Tube Green	Right	B, D, C, E, A	Tube Grey + Tupp. Green
Berci	Old	Female	Left	E, C, D, A, B	Tupp. Pink + Tube Green	Right	A, B, D, E, C	Tube Grey + Tupp. Green
Carlos	Old	Female	Right	A, B, E, D, C	Tupp. Green + Tube Grey	Left	E, B, A, D, C	Tube Green + Tupp. Pink
Dió	Old	Female	Right	B, E, C, D, A	Tupp. Green + Tube Grey	Left	D, E, B, C, A	Tube Green + Tupp. Pink
Scooby	Old	Male	Right	C, D, E, B, A	Tupp. Green + Tube Grey	Left	C, D, B, A, E	Tube Green + Tupp. Pink
Mackó	Old	Male	Right	D, C, A, B, E	Tupp. Green + Tube Grey	Left	B, C, D, E, A	Tube Green + Tupp. Pink
Lili	Old	Male	Right	E, C, A, D, B	Tupp. Green + Tube Grey	Left	A, C, E, B, D	Tube Green + Tupp. Pink

Bizsu	Young	Female	Left	E, D, A, B, C	Tube Grey + Tupp. Green	Right	A, D, B, C, E	Tupp. Pink + Tube Green
Mogyoró	Young	Female	Left	D, C, E, A, B	Tube Grey + Tupp. Green	Right	B, D, A, E, C	Tupp. Pink + Tube Green
Dingó	Young	Male	Left	C, A, E, B, D	Tube Grey + Tupp. Green	Right	C, A, D, E, B	Tupp. Pink + Tube Green
Bogi	Young	Male	Left	B, D, C, A, E	Tube Grey + Tupp. Green	Right	D, C, A, E, B	Tupp. Pink + Tube Green
Mala	Young	Male	Left	A, D, B, E, C	Tube Grey + Tupp. Green	Right	E, D, C, A, B	Tupp. Pink + Tube Green
Szőrmi	Young	Male	Right	E, D, B, C, A	Tube Green + Tupp. Pink	Left	A, B, E, D, C	Tupp. Green + Tube Grey
Gomez	Young	Male	Right	D, E, B, C, A	Tube Green + Tupp. Pink	Left	B, A, C, E, D	Tupp. Green + Tube Grey
DióE	Young	Male	Right	C, A, D, B, E	Tube Green + Tupp. Pink	Left	C, E, B, D, A	Tupp. Green + Tube Grey
Zafír	Young	Female	Right	B, D, C, A, E	Tube Green + Tupp. Pink	Left	D, A, C, E, B	Tupp. Green + Tube Grey
Kócos	Young	Female	Right	A, C, B, E, D	Tube Green + Tupp. Pink	Left	E, D, B, A, C	Tupp. Green + Tube Grey
Popeye	Old	Male	Left	E, D, C, B, A	Tube Grey + Tupp. Green	Right	A, D, C, E, B	Tupp. Pink + Tube Green
Buch	Old	Male	Left	D, A, C, E, B	Tube Grey + Tupp. Green	Right	B, A, E, D, C	Tupp. Pink + Tube Green
Lupi	Old	Female	Left	C, A, E, B, D	Tube Grey + Tupp. Green	Right	C, D, E, B, A	Tupp. Pink + Tube Green
Zsoli	Old	Female	Left	B, D, A, C, E	Tube Grey + Tupp. Green	Right	D, E, C, B, A	Tupp. Pink + Tube Green
Tala	Old	Female	Right	E, B, D, C, A	Tube Green + Tupp. Pink	Left	A, C, D, B, E	Tupp. Green + Tube Grey
Smafu	Old	Female	Right	D, E, A, B, C	Tube Green + Tupp. Pink	Left	B, C, E, A, D	Tupp. Green + Tube Grey
Panda	Old	Female	Right	C, B, E, A, D	Tube Green + Tupp. Pink	Left	C, D, E, A, B	Tupp. Green + Tube Grey
Cheyenne	Old	Male	Right	B, D, C, A, E	Tube Green + Tupp. Pink	Left	D, B, E, C, A	Tupp. Green + Tube Grey

Table S3. Control trials comparison for the Memory test

Trials	Proportion of success		W	*p*
	**Retest**	**Control**		
**Trial 1**	47%	16%	168	0.008
**Trial 2**	55%	5%	190	< 0.001
**Trial 3**	45%	8%	119	0.003

Table S4. Inter-observer agreement

Variable	Agreement	p-values
**Activity level**	Cohen's kappa = 1.00	0.005
**Social interaction**	Cohen's kappa = 1.00	< .001
**Object manipulation**	Cohen's kappa = 1.00	< .001
**Neophilic behaviour**	Cohen's kappa = 1.00	< .001
**Novel Object duration (left)[a]**	Spearman's rho = 1.00	
**Novel Object duration (right)[a]**	Spearman's rho = 1.00	
**Errors (trial 1)**	Cohen's kappa = 1.00	< .001
**Errors (trial 2)**	Cohen's kappa = 1.00	< .001
**Errors (trial 3)**	Cohen's kappa = 1.00	< .001
**Errors (trial 4)**	Cohen's kappa = 1.00	< .001
**Errors (trial 5)**	Cohen's kappa = 1.00	< .001
**Object interaction**	Spearman's rho = 1.00	
**Object avoidance**	Spearman's rho = 1.00	

[a]These variables were used to calculate the recognition index
